# 516. Evaluation of COVID-19 Monoclonal Antibody Therapies for the Treatment of Non-hospitalized Patients with COVID-19

**DOI:** 10.1093/ofid/ofab466.715

**Published:** 2021-12-04

**Authors:** Faiza Morado, Neha Nanda

**Affiliations:** 1 Keck Medical Center of USC, Pasadena, CA; 2 Keck School of Medicine, Los Angeles, CA

## Abstract

**Background:**

In an effort to reduce strain on healthcare systems with patient hospitalizations and deaths due to COVID-19, the US Food and Drug Administration (FDA) issued an Emergency Use Authorization (EUA) for 2 monoclonal antibodies for the treatment of COVID-19 in November 2020: bamlanivimab (BAM) and casirivmab-imdevimab (CAS-IMD). While clinical trial data demonstrated reductions in hospitalization rate, real-world data at the time of approval was vastly limited.

**Methods:**

A retrospective chart review of non-hospitalized patients who received either BAM or CAS-IMD from November 27^th^, 2020 to February 16^th^, 2021. Variables included timing of monoclonal antibody infusion, adverse events, and 30-day hospitalization rate. Descriptive statistics were calculated for all data.

**Results:**

101 patients received either BAM (75.2%) or CAS-IMD (24.8%) at a median of 6 days (IQR 4-7) from reported symptom onset. The most commonly reported symptoms of COVID-19 at time of referral were cough (57.4%), fever (29.7%), and myalgia (27.7%). All patients (100%) had at least 1 documented EUA defined risk factor for severe COVID-19 (Table 1). Following transfusion, 7/101 (6.9%) and 3/101 (3.0%) experienced mild to moderate and severe adverse events, respectively (Table 2). At day 30, 5 patients (5.0%) were hospitalized with COVID-19 at a median of 7 days (IQR 3-8) post monoclonal antibody infusion.

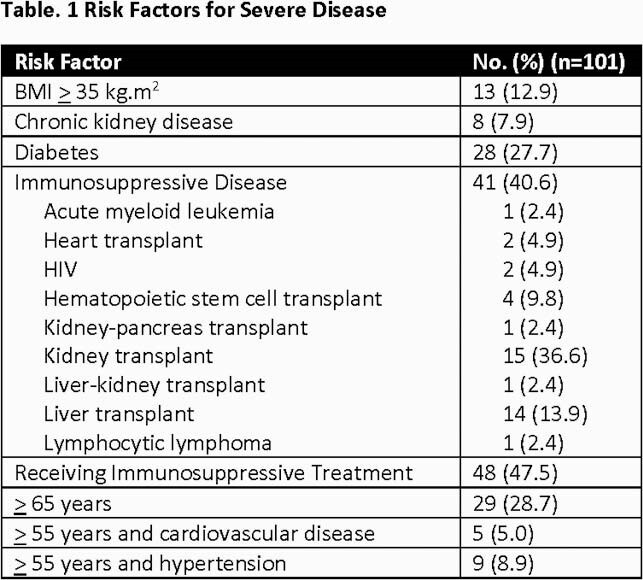

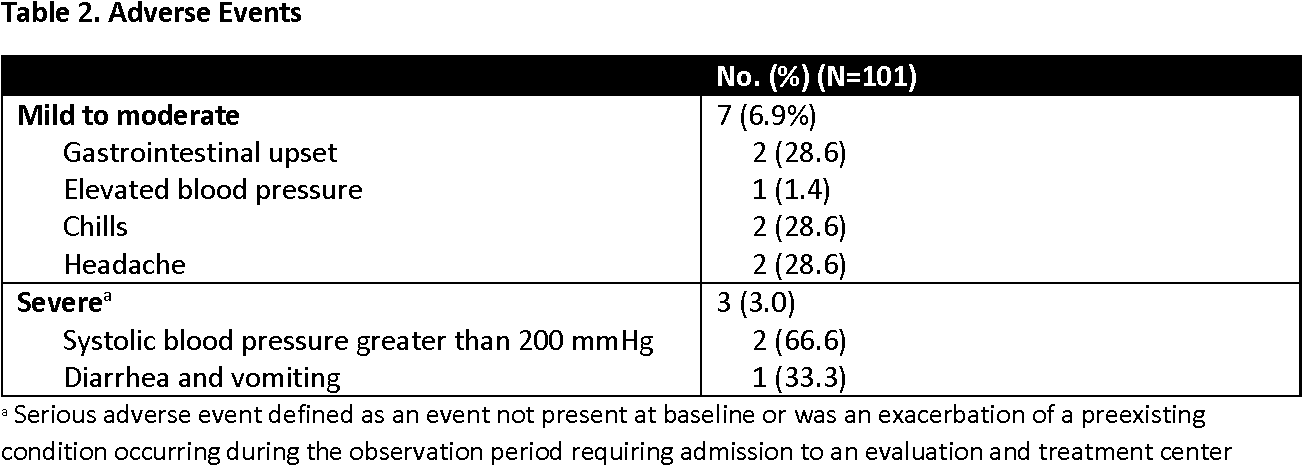

**Conclusion:**

We observed a higher frequency of hospitalization compared to 1.6% for BAM in BLAZE-1 and 3% for CAS-IMD in REGN-COV-2. This observation may reflect our higher risk population as all patients presented with at least 1 risk factor for severe disease compared to 69.6% and 65.0% in BAM and CAS-IMD clinical trials, respectively. Additionally, patients presented with longer durations of symptoms prior to infusion in our study population compared to 3 days reported in BAM and 4 days reported in CAS-IMD trials. Since the conclusion of this study, the FDA revoked the EUA for BAM administered alone based on increased observations of resistant variants to BAM monotherapy. However, our observations highlight the need for further exploration in the prevention of hospitalization in high risk populations as well as the optimal timing of monoclonal antibody therapy.

**Disclosures:**

**All Authors**: No reported disclosures

